# Mechanisms of charge transfer and electronic properties of Cu_2_ZnGeS_4_ from investigations of the high-field magnetotransport

**DOI:** 10.1038/s41598-017-10883-0

**Published:** 2017-09-06

**Authors:** Maxim Guc, Erkki Lähderanta, Elena Hajdeu-Chicarosh, Sergiu Levcenko, Mikhail A. Shakhov, Ivan Zakharchuk, Ernest Arushanov, Konstantin G. Lisunov

**Affiliations:** 1 Department of Mathematics and Physics, Lappeenranta University of Technology, PO Box 20, FIN-53851 Lappeenranta, Finland; 2 Institute of Applied Physics, Academy of Sciences of Moldova, Academiei Str. 5, MD-2028 Chisinau, Republic of Moldova; 3 Helmholtz Zentrum Berlin für Materialien und Energie, Hahn-Meitner-Platz 1, D-14109 Berlin, Germany; 4 Ioffe Institute, Politehnicheskaya Str. 26, St, Petersburg, 194021 Russian Federation

## Abstract

Recent development of the thin film solar cells, based on quaternary compounds, has been focused on the Ge contain compounds and their solid solutions. However, for effective utilization of Cu_2_ZnGeS_4_, deeper investigations of its transport properties are required. In the present manuscript, we investigate resistivity, ρ (*T*), magnetoresistance and Hall effect in p-type Cu_2_ZnGeS_4_ single crystals in pulsed magnetic fields up to 20 T. The dependence of ρ (*T*) in zero magnetic field is described by the Mott type of the variable-range hopping (VRH) charge transfer mechanism within a broad temperature interval of ~100–200 K. Magnetoresistance contains the positive and negative components, which are interpreted by the common reasons of doped semiconductors. On the other hand, a joint analysis of the resistivity and magnetoresistance data has yielded series of important electronic parameters and permitted specification of the Cu_2_ZnGeS_4_ conductivity mechanisms outside the temperature intervals of the Mott VRH conduction. The Hall coefficient is negative, exhibiting an exponential dependence on temperature, which is quite close to that of ρ(*T*). This is typical of the Hall effect in the domain of the VRH charge transfer.

## Introduction

Recent development of the solar cell science and technology, based on the I_2_-II-IV-VI_4_ quaternary chalcogenides, has been focused on their solid solutions with a partial cation substitution. The most promising results have been achieved with Cu_2_ZnSn_x_Ge_1−x_(S,Se)_4_ belonging to this family of compounds^1–5^. Herewith, even introduction of a small amount of Ge as dopant in the Cu_2_ZnSnSe_4_ layers (with Ge contents well below 10%) yields a significant increase of the solar cell efficiency^4, 5^. Following this tendency, an incrementing amount of investigations of the Cu_2_ZnSn_x_Ge_1-x_(S,Se)_4_ solid solutions has been published in recent time^6–12^. On the other hand, an alternative way for a future development of the solar cells based on quaternary compounds is considered widely nowadays. It deals with tandem solar cells, where quaternary compounds are used in a top device with a higher band gap^13–15^. Theoretical investigations predict an inverse dependence of the top device efficiency on the absorbing layer band gap^13^. Accordingly, an absorbing layer with the band gap, *E*_g_, of 2 eV or higher requires less than 9% of efficiency for the top solar cell to ensure more than 25% of total efficiency of the tandem device. In this sense, Cu_2_ZnGeS_4_ (CZGeS) is a good candidate for utilization. Indeed, its band gap lies in the range of *E*_g_ ~2.1–2.3 eV^16–20^, whereas the absorption coefficient exceeding 10^4^ cm^-1^ ^19^ is an additional advantage. Besides of the photovoltaic applications, CZGeS shows promising results also for the hydrogen evolution of water^21^ and is regarded as a possible application in thermoelectric materials^22^. However, additional investigations of the fundamental properties of CZGeS should be performed, before its effective utilization in different optoelectronic devices would be possible, including a solid solution or the compound itself.

The main feature of CZGeS, distinguishing it from other quaternaries, is existence of the two different structural types, kesterite^6, 10, 12, 23–26^ and wurtzstannite^17, 18, 20, 27, 28^, both observed experimentally. Along with the structural investigations^17, 18, 23, 24, 27^, the optical^16–20^ and vibrational^10, 12, 25, 26, 28^ properties of CZGeS have been studied extensively for both types. On the other hand, the electronic transport of CZGeS, which is important for any material utilization in optoelectronic devices, is still investigated quite insufficiently. There exist only a few articles, where the room temperature resistivity^16, 17, 20^, and its behavior in the high temperature range of 450–550 K^22, 29^ have been observed, without any analysis of the data. Probably, the only exclusion is an explicit analysis of the resistivity and magnetoresistance in the kesterite type Cu_2_ZnSn_x_Ge_1-x_Se_4_ solid solutions^11^.

In the present work, the temperature dependence of the zero-field resistivity, ρ(*T*), and magnetotransport in CZGeS single crystals with wurtzstannite structure are investigated within a wide interval below 300 K. This permits identification of the conductivity mechanisms in different temperature ranges and determination of important electronic parameters, which are required to estimate efficiency of the material in optoelectronic devices. In particular, the joint analysis of the resistivity and magnetoresistance data has been widely demonstrated to be an effective method for such a purpose (see e. g. refs 30–38 and references therein), which has been already utilized recently in various kesterite type quaternary compounds^11, 39^.

## Results and Discussion

### Experimental results

**Figure 1:**
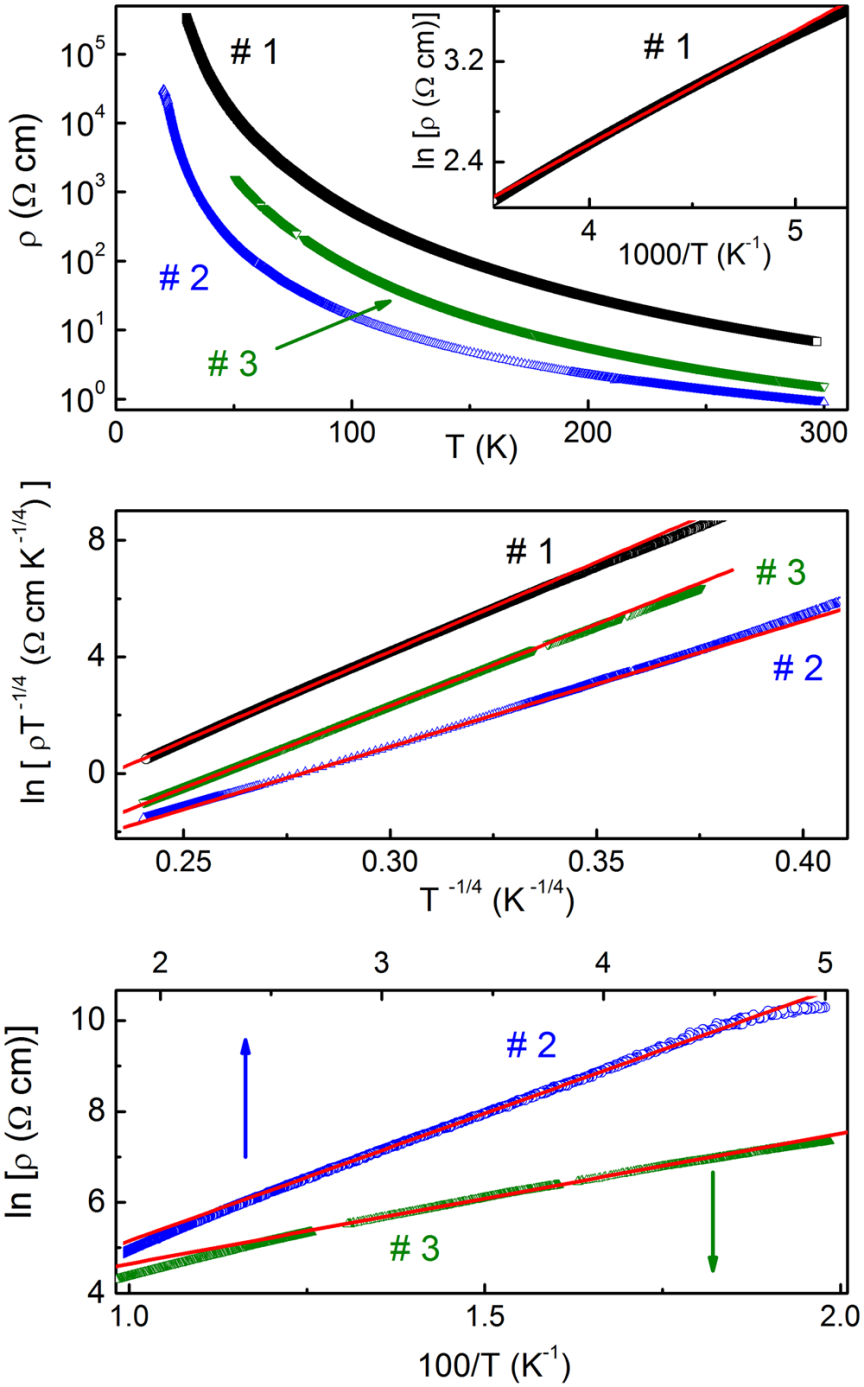
Temperature dependence of the resistivity (top panel), the plots of ln (ρ *T* ^−1/4^) vs. *T* ^−1/4^ (middle panel) and the plots of ln ρ vs. *T* ^−1^ (inset to the top panel and bottom panel) for samples #1, #2 and #3. The lines are linear fits.

As can be seen in the top panel of Fig. 1, ρ(*T*) of all samples is similar, exhibiting a strongly activated behavior within the whole temperature range, available for the resistivity measurements.

**Figure 2:**
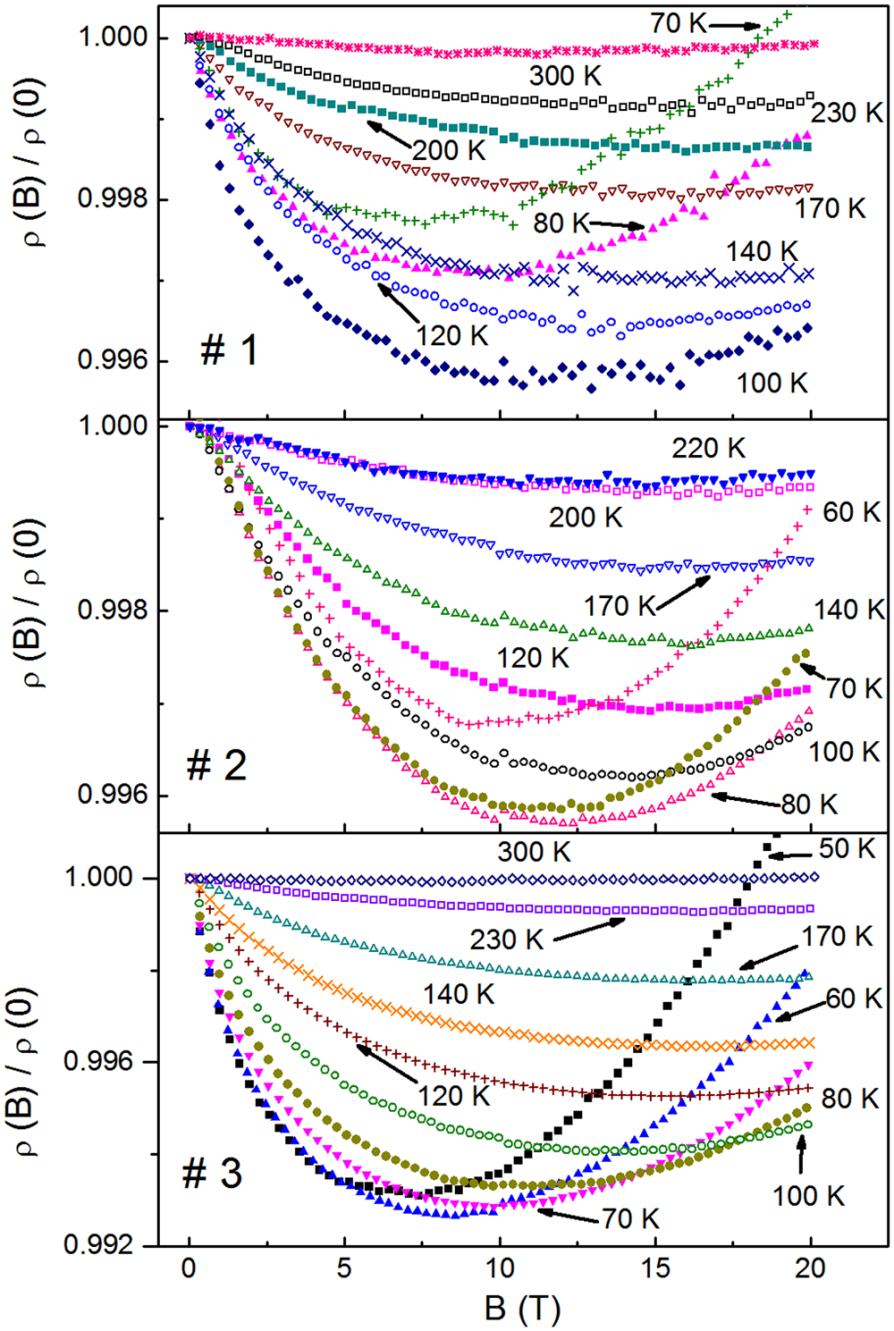
The dependence of the relative resistivity, ρ(*B*)/ρ(0), on *B* for the investigated samples at different temperatures.

As follows from Fig. 2, magnetoresistance (MR) contains both the positive (pMR) and the negative (nMR) contributions within the whole temperature range of ~50–300 K. In particular, the relative MR, Δρ/ρ ≡ [ρ(*B*) − ρ(0)]/ρ(0), is negative almost at any *B* and *T*, excluding only the cases of *T* = 70 K in #1, and *T* = 50 K and 300 K in #3 at *B* > 18 T. The behavior of MR with increasing temperature is noticeable, having different temperature dependences within two different temperature intervals, addressed to the lower and higher *T*, as well as various shapes of the curves in Fig. 2 in the corresponding intervals. Namely, between *T* = 70–100 K, 60–80 K and 50–70 K in #1, #2 and #3, respectively, the MR effect increases with increasing *T*. The decay of MR with *T*, conventional for semiconductors, starts only with further increase of the temperature beyond the intervals above. In addition, such a transition is accompanied by a gradual flattening of the MR shape, showing different relations between temperature dependences of the nMR and pMR contributions in each of the temperature intervals, defined above.

**Figure 3:**
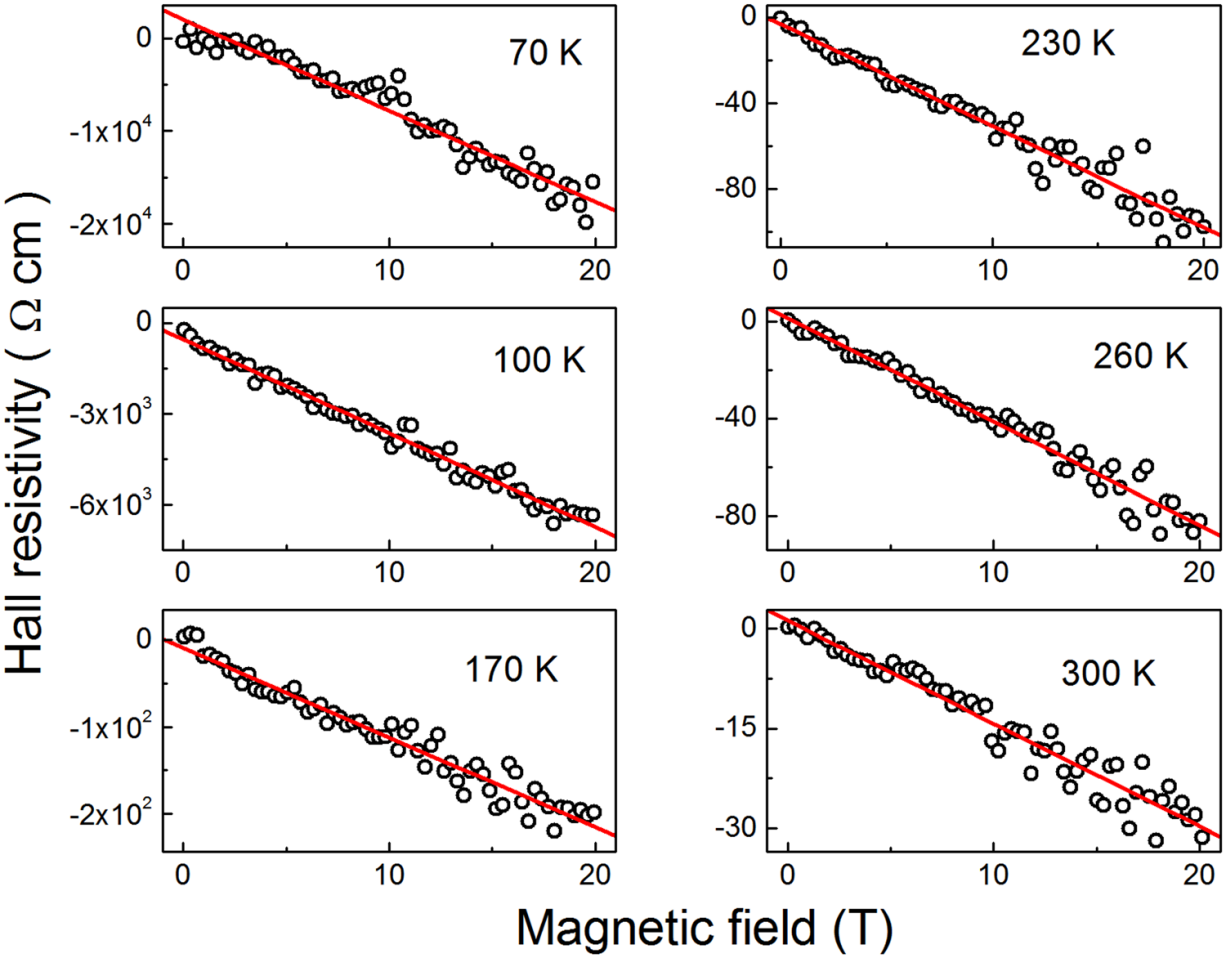
The dependence of the Hall resistivity on the magnetic field for sample #1 at different temperatures. The lines are linear fits.

In Fig. 3 is evident, that the Hall resistivity, ρ_H_, of sample #1 is negative at any *T* between 70–300 K, despite of the positive sign of the thermopower and the p-type conduction of our CZGeS samples. Note, that the hole conduction is typical of all Cu based quaternary compounds of this family of materials, although possessing the kesterite structure (see e. g. refs 11, 39 and references therein). One cannot observe distinguishable deviations of the ρ_H_(*B*) function from linearity, which, however, may be connected partially to a rather high scattering of the data points in Fig. 3. On the other hand, a strong dependence of ρ_H_(*T*) between *T* = 70 and 230 K is evident at any field, weakening substantially with further increase of *T* up to 300 K.

### Temperature dependence of resistivity at B = 0

In the quaternary Cu based kesterite materials the hopping charge transfer, mainly the Mott variable-range hopping (VRH), has been reported for different chemical content, stoichiometry and macroscopic state, including single crystals^11, 39–42^, thin polycrystalline films^43–48^ and powder samples^49, 50^. The temperature border of the hopping conduction is usually very high, stretching even up to the room temperature already in single crystals, whereas width of the defect acceptor band, *W*, vary from ~10 to ~100 meV and even higher^39–50^. Both these features are quite uncommon for conventional semiconductors with shallow hydrogenic impurities, exhibiting usually *W* between ~1–10 meV and the hopping charge transfer at temperatures well below 300 K^32–36, 51–54^. The point is that the energy of the main (most stable) acceptor level, *E*_A_, connected to Cu_Zn_ antisites^55–60^, in such materials is rather high (e.g. in Cu_2_ZnSnS_4_ and Cu_2_ZnSnSe_4_ the values of *E*_A_ ≈ 120–140 meV have been reported^39, 55–58^), which favors the hopping charge transfer in general. On the other hand, domination of the Mott VRH conduction is connected with a high intrinsic lattice disorder, caused by formation of the disordered kesterite phase^61–63^. In turn, the disorder can be effectively varied experimentally, as has been demonstrated by observations of considerable changes in *W* of the Cu_2_ZnSnS_4_ films after thermal annealing^44^. At this point, the VRH conduction is quite expectable in CZGeS, too, supported by *E*_A_ = 140 meV^64^ and by a complicated crystal structure^23–28^ permitting similar reasons for the microscopic disorder, as in kesterites^61–63^.

Therefore, it is reasonable to start the analysis of ρ(*T*) in our samples by searching the possible interval of the Mott VRH charge transfer, given by the resistivity law

1 $${\rm{\rho }}(T)=D{T}^{1/4}\exp [{(\frac{{T}_{0}}{T})}^{1/4}].$$

Here, *D* is the VRH prefactor constant and

2 $${T}_{0}={\rm{\beta }}{[{k}_{{\rm{B}}}g({\rm{\mu }}){a}^{3}]}^{-1}$$

is the VRH characteristic temperature, where *k*_B_ is the Boltzmann constant, β = 21 is a numerical constant, *g*(μ) is the density of the localized states (DOS) at the Fermi level, μ, and *a* is the localization radius of charge carriers, scaling the space decay of the impurity wave functions^51–54^. Generally, the VRH conduction sets in, when it is energetically more profitable for a carrier to jump beyond nearest centers, with levels with large energy difference, towards more distant centers with a smaller energy difference with respect to the initial center^52^. Therefore, the factors favoring the VRH charge transfer are connected to decreasing temperature and increasing degree of the disorder. Formally, the VRH conduction of Eq. (1) is expected at ε_m_(*T*) < *W*, where ε_m_(*T*) ≈ 0.5 *k*_B_ (*T* ^3^ *T*_0_)^1/2^ is the mean hopping energy^40, 53^. In addition, Eqs (1) and (2) have been obtained in refs 53 within a percolation approach for the shallow hydrogenic levels, satisfying the condition of *E*_A_/*E*_g_ <<1. With the values of *E*_g_ ~2.1–2.3 eV and *E*_A_ = 140 meV, given above, one finds *E*_A_ /*E*_g_ ~0.06–0.07, in agreement with the condition above.

Indeed, the plots in the middle panel of Fig. 1 exhibit broad linear intervals, pertinent to the Mott VRH conduction according to Eq. (1). The values of *T*_0_ have been found with the slopes of these plots. The temperature intervals, Δ*T*_v_, of the Mott VRH charge transfer, have been obtained with the linear intervals of the corresponding plots. The data of *T*_0_ and Δ*T*_v_ are collected in Table 1. In addition, *W* in Table 1 has been evaluated with the expression of *W* ≈ 0.5 *k*_B_ (*T*_v_^3^ *T*_0_)^1/4^ ^40, 53^, where *T*_v_ is the Mott VRH onset temperature on cooling (i. e. the right border of the intervals Δ*T*_v_ in Table 1).

**Table 1:** The VRH conduction interval (Δ*T*_v_), VRH characteristic temperature (*T*_0_), width of acceptor band (*W*), and MR coefficient (*A*_04_).

Sample	Δ*T*_v_ K	*T*_0_ 10^7^ K	*W* meV	*A*_04_ 10^−3^ T ^−2^ K^1/4^
#1	95–210	1.43 ± 0.02	146 ± 6	1.11 ± 0.03
#2	90–195	0.343 ± 0.003	97 ± 4	1.10 ± 0.02
#3	95–185	1.00 ± 0.01	121 ± 5	1.73 ± 0.03

### Density of the acceptor states

**Figure 4:**
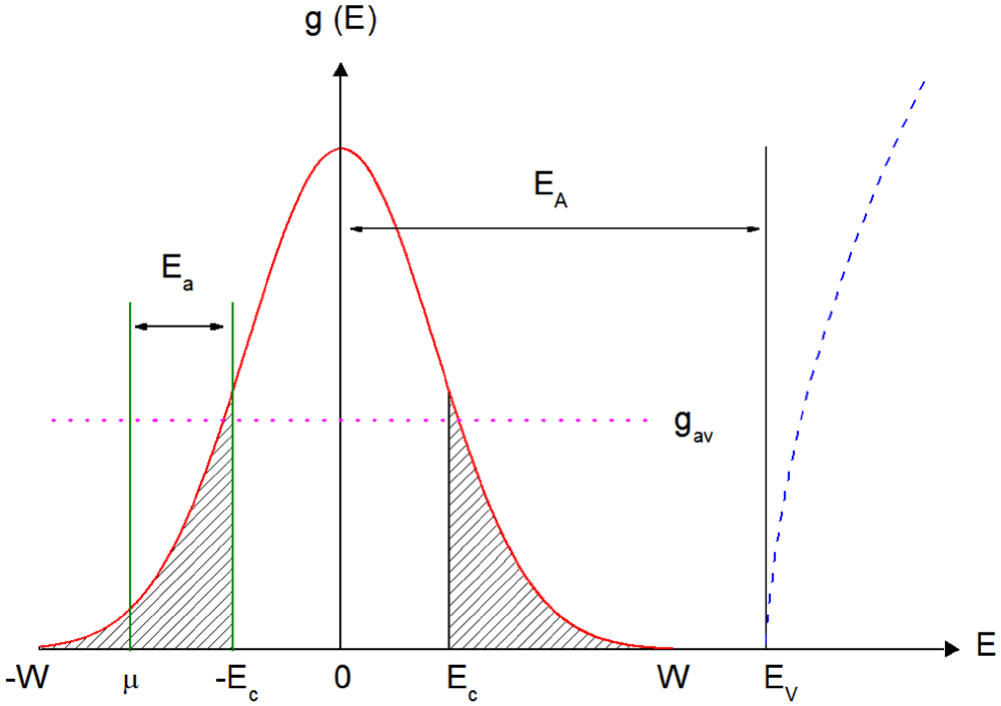
The DOS of the acceptor band (schematically). The localized states are hatched. *E*_v_ is the valence band edge (given by the dashed line). The dotted line is the average DOS value, *g*_av_.

In order to proceed the analysis of the resistivity and MR, below we assume a conventional DOS of the Anderson type^51, 52^ in the acceptor band, exhibited in Fig. 4. It is considered to be symmetrical, centered at *E*_A_ = 0 and is characterized by a finite total width 2*W*. The parameters −*E*_c_ and *E*_c_ in Fig. 4 are the mobility edges, separating the delocalized acceptor states from the localized states of the acceptor band, which are hatched. Finally, a small overlap of the acceptor band with the states of the valence band, taking place for #1 (in Table 1, *W* = 146 meV exceeds slightly *E*_A_ = 140 meV^64^) can be neglected in the analysis below. Indeed, the position of μ is expected to lie among the localized states, because otherwise the conductivity would be metallic^51, 52^ contradicting to the activated behavior of ρ(*T*) in Fig. 1. Such position should be chosen near the left edge of the DOS, as shown in Fig. 4. This corresponds to a sufficiently high degree of compensation, *K* = *N*_D_/*N*_A_, where *N*_A_ is the acceptor concentration and *N*_D_ is the concentration of the compensating donors. Therefore, the details of the energy spectrum on the right edge of the DOS, which is close to the valence band edge, are unimportant. In turn, this position of μ is connected with the high *W* values, which are comparable with the *E*_A_ value (see Table 1). This excludes the possibility of μ lying among the right-hand interval of the localized states in Fig. 4, because in this case the conductivity, connected to the activation of the holes from μ to the valence band states, would dominate at any *T* due to a small difference between *W* and *E*_A_. Instead, another type of the resistivity^51, 52^,

3 $${\rm{\rho }}(T)={{\rm{\rho }}}_{0}\exp [\frac{{E}_{a}}{{k}_{B}T}],$$

characterized by the constant prefactor ρ_0_ and the activation energy

4 $${E}_{a}=|{\rm{\mu }}-{E}_{c}|,$$

has been predicted for the position of μ shown in Fig. 4. Namely, as outlined in Fig. 4, the charge transfer connected to Eq. (3) is determined by activation of the localized holes from μ to −*E*_c_, or, more strictly, from the localized states below the Fermi level into the interval of delocalized states of the impurity band^51, 52^. In turn, the temperature interval of Eq. (3), Δ*T*_a_, may lie either above or below that of the VRH interval Δ*T*_v_, depending on the distance between μ and *–E*_c_ according to Eqs (3) and (4). In particular, both cases of the position of Δ*T*_a_ with respect to Δ*T*_v_ have been observed in kesterites^39, 41, 50^.

### Analysis of magnetoresistance

In doped semiconductors pMR in the Mott VRH conduction regime is connected mainly with the shrinkage of the impurity wave functions by the magnetic field^53^. In weak magnetic fields of λ(*B*)>> *a*, where λ(*B*) = [ħ/(*eB*)]^1/2^ is the magnetic length (here, ħ is the Planck constant and *e* is the elementary charge), pMR of this mechanism can be expressed as^53^

5 $$\mathrm{ln}[\frac{{\rm{\rho }}\,(T,B)}{{\rm{\rho }}\,(T,0)}]={A}_{4}(T){B}^{2},$$

where $${A}_{4}(T)={A}_{04}{T}^{-3/4}$$ and

6 $${A}_{04}={t}_{4}({e}^{2}{a}^{4}/{\hslash }^{2}){T}_{0}^{3/4}.$$

Here, *t*_4_ = 5/2016 is a numerical constant^53^. The most general mechanism of nMR, proposed for the Mott VRH charge transfer, is addressed to the quantum interference of the direct paths, arising from multiple scattering of the hopping charge carriers by the intermediate centers of the same nature as those involved in the hopping process (acceptors in the case of CZGeS)^65–69^. This mechanism has been proposed initially for the VRH conduction at sufficiently low temperatures, where the inelastic collision frequency is expected to be also low, providing conservation of a carrier phase coherence during multiple scattering^65^. However, in strongly disordered systems the constraint above may be weakened, which expands the temperature interval, where the interference effects are important. The point is that a higher disorder, being eventually a main reason of the interference effects^65–69^, would lead to their stronger contribution to MR at the same *T*. Therefore, one can expect a corresponding increase of the upper temperature limit, where the interference effects still survive, when the disorder is increased. Indeed, nMR in the VRH regime, attributable to the interference mechanism above, has been observed at *T* up to 300 K in disordered polyaniline silicon nanocomposites^70^ and highly disordered pregraphitic carbon nanofiber^71^, as well as up to 280 K in Cu_2_Zn(Sn_x_Ge_1-x_)Se_4_ single crystals^11^.

As mentioned above, CZGeS and related quaternary compounds are characterized, generally, by high intrinsic lattice disorder. In particular, such disorder leads to a considerable broadening of the impurity band, which exceeds substantially typical values of *W* in doped semiconductors. As can be seen in Table 1, large values of *W* characterize our CZGeS samples, too. This permits us to use tentatively the orbital interference mechanism^65–69^ to interpret the nMR effect in our material.

For the weak scattering, given by the relation of

7 $${A}_{s}\ll 1,$$

where *A*_s_ ≈ π*E*_A_β/(3*k*_B_ *T*_0_) × (*T*_0_/*T*)^1/4^ ^36, 68^, and below a critical field *B*_c_, nMR can be expressed as Δρ/ρ = −*a*_2_(*T*)*B* ^2^, whereas above *B*_c_ it is given by the expression Δρ/ρ = −*a*_1_(*T*)*B* ^68^. Here, *a*_2_(*T*) ∝ *T* ^−1^, *a*_1_(*T*) ∝ *T* ^−3/4^ and

8 $${B}_{c}\approx {(\Delta /{E}_{A})}^{1/2}{B}_{{\rm{q}}},$$

where Δ = *k*_B_(*T* ^3^ *T*_0_)^1/4^, *B*_q_ ≈ 2^2/3^πħ(*E*_A_ *k*_B_ *T*)^1/2^/(*eJ*_0_ *a* ^2^) × (*T*/*T*_0_)^1/4^ and *J*_0_ is the prefactor of the overlap integral^68^. Finally, at *B* > *B*_c_′, where *B*_c_′ is defined by the condition of λ^2^(*B*_c_′) ≈ *R*_h_ *a* and *R*_h_ ≈ γ_4_ *a*(*T*_0_/*T*)^1/4^ is the mean hopping length (where γ_4_ ≈ 0.357 is a constant^72^), the nMR law of Δρ/ρ ∝ *B* ^1/2^ has been predicted^69^.

First, we estimate the value of *B*_c_′ for our CZGeS samples. As evident in the top panel of Fig. 1, the strongly activated behavior of ρ(*T*) is consistent with the state of all the samples lying relatively far from the metal-insulator transition, meaning that the value of *a* should exceed only slightly the value of the Bohr radius, *a*_B_ = ħ^2^κ_0_/(*me* ^2^). For evaluation of *a*_B_, the theoretical values of the dielectric constant far from the metal-insulator transition, κ_0_ = 6.8^73^, and of the mean hole effective mass of a stannite CZGeS, *m* = 0.48*m*_0_ (where *m*_0_ is the free electron mass)^74^ can be used, yielding *a*_B_ = 7.5 Å. This result is supported completely by the value of *E*_A_ = ħ^2^/(2*ma*_B_^2^) = 140 meV, coinciding with its value observed experimentally in ref. 64. Hence, taking e. g. *a* = 10 Å, we obtain with the direct expression of *B*_c_′ = ħ/(*a* ^2^ *e*γ_4_) × (*T*/*T*_0_)^1/4^, the values of *B*_c_′ ≈ 100–140 T at *T* = 100 K and 110–160 T at *T* = 200 K. These data permit to exclude a possibility of the square-root dependence of nMR on *B* above from further consideration, meaning that *B*_c_′ exceeds considerably the maximum applied field of *B* = 20 T irrespective the possible error of the *a* value used above. Similarly the magnetic field *B*_2_, addressed to violation of Eq. (5) and defined by the condition of λ(*B*_2_) ~ *a*, i. e. *B*_2_ ~ ħ/(*ea* ^2^), can be estimated to lie close to *B*_2_ ~600 T. This guarantees applicability of Eq. (5) for our samples, too.

Eventually, the data of *A*_s_ ≈ 0.040–0.049, 0.12–0.14 and 0.055–0.064 can be evaluated for #1, #2 and #3, respectively, with the expression of *A*_s_, given below Eq. (7) directly, using *E*_A_ = 140 meV and the values of *T*_0_ and Δ*T*_v_ from Table 1. This guarantees the regime of the weak scattering in agreement with Eq. (7). However, the value of the critical field *B*_c_ dividing the field intervals of the quadratic and linear nMR dependences above cannot be estimated beforehand requiring the value of the parameter *J*_0_. On the other hand, as evident in Fig. 2, the field interval of Δρ/ρ ∝ *B* ^2^ in our samples looks to be quite narrow and is shifted to the low fields (lying probably below *B* ~1–2 T), constituting only a negligible part of the investigated field interval, where the MR data are less confident than at higher *B*. Therefore, it is more consistent to concentrate the analysis of MR on the higher field interval of *B* > 10 T, where the linear field dependence of nMR and the quadratic dependence of the pMR on *B* are more expected to persist. Hence, taking into account the consideration of nMR above, Eq. (5) for pMR and the evident fact that the expression of ln[ρ (*T*, *B*)/ρ (*T*, 0)] ≈ Δρ/ρ is fulfilled with a high accuracy, provided that the relative MR does not exceed 1% anywhere (Fig. 2), we can use for the analysis of MR the expression

9 $${\rm{\Delta }}{\rm{\rho }}/{\rm{\rho }}+{a}_{{\rm{1}}}(T)B={A}_{4}(T){B}^{2}.$$

**Figure 5:**
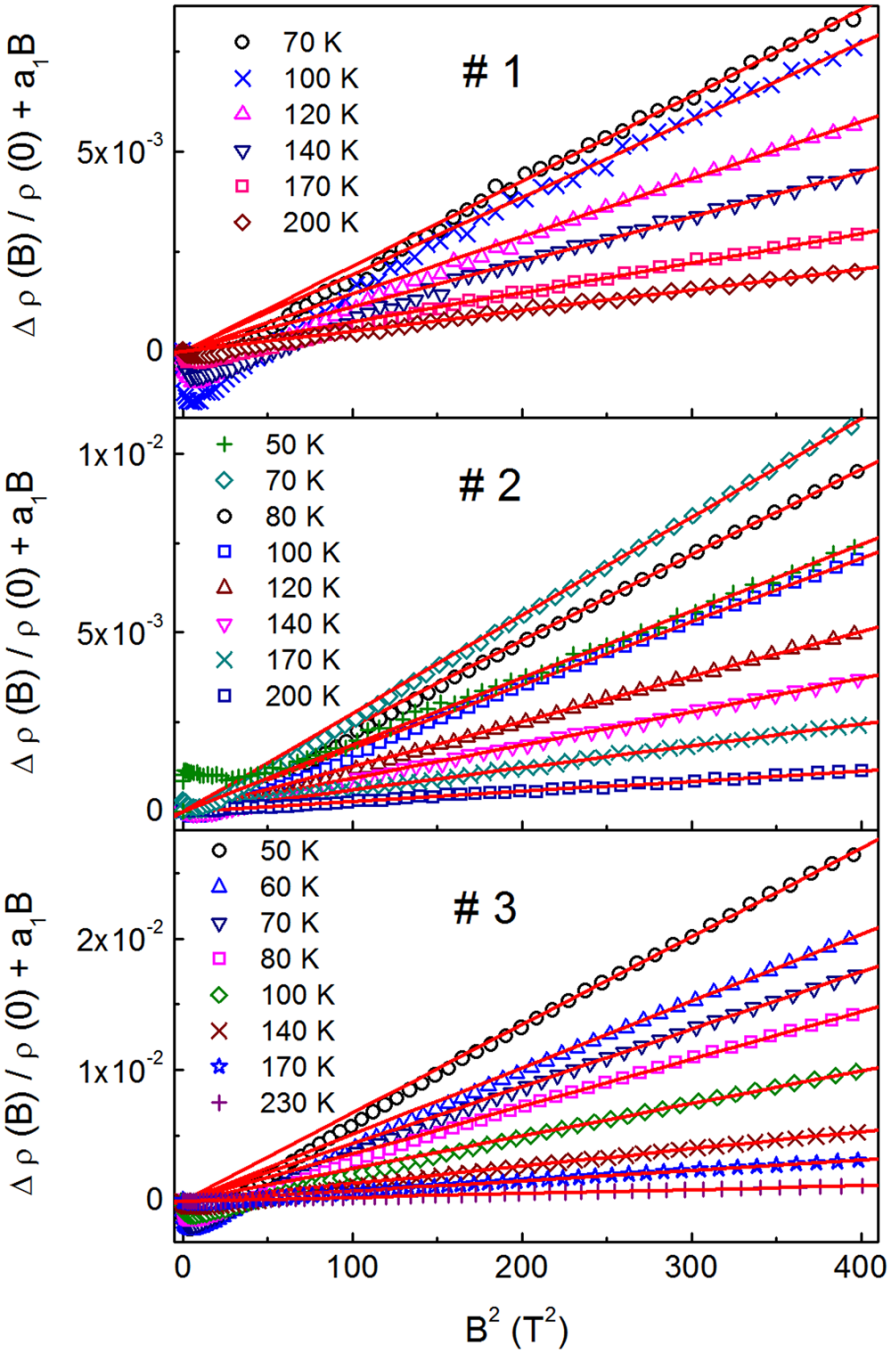
The plots of Δρ(*B*)/ρ(0) + *a*_1_ *B* vs. *B* ^2^ for the investigated samples at different temperatures. The lines are linear fits according to Eq. (9).

The values of *a*_1_ (*T*) are found by plotting the left-hand side of Eq. (9) vs. *B* ^2^ and choosing *a*_1_(*T*) to obtain the best linearization of the plots. This is done by minimizing the standard deviation (SD), provided that all the plots should pass through the origin. Finally, the slope of these plots in Fig. 5 gives *A*_4_(*T*).

**Figure 6:**
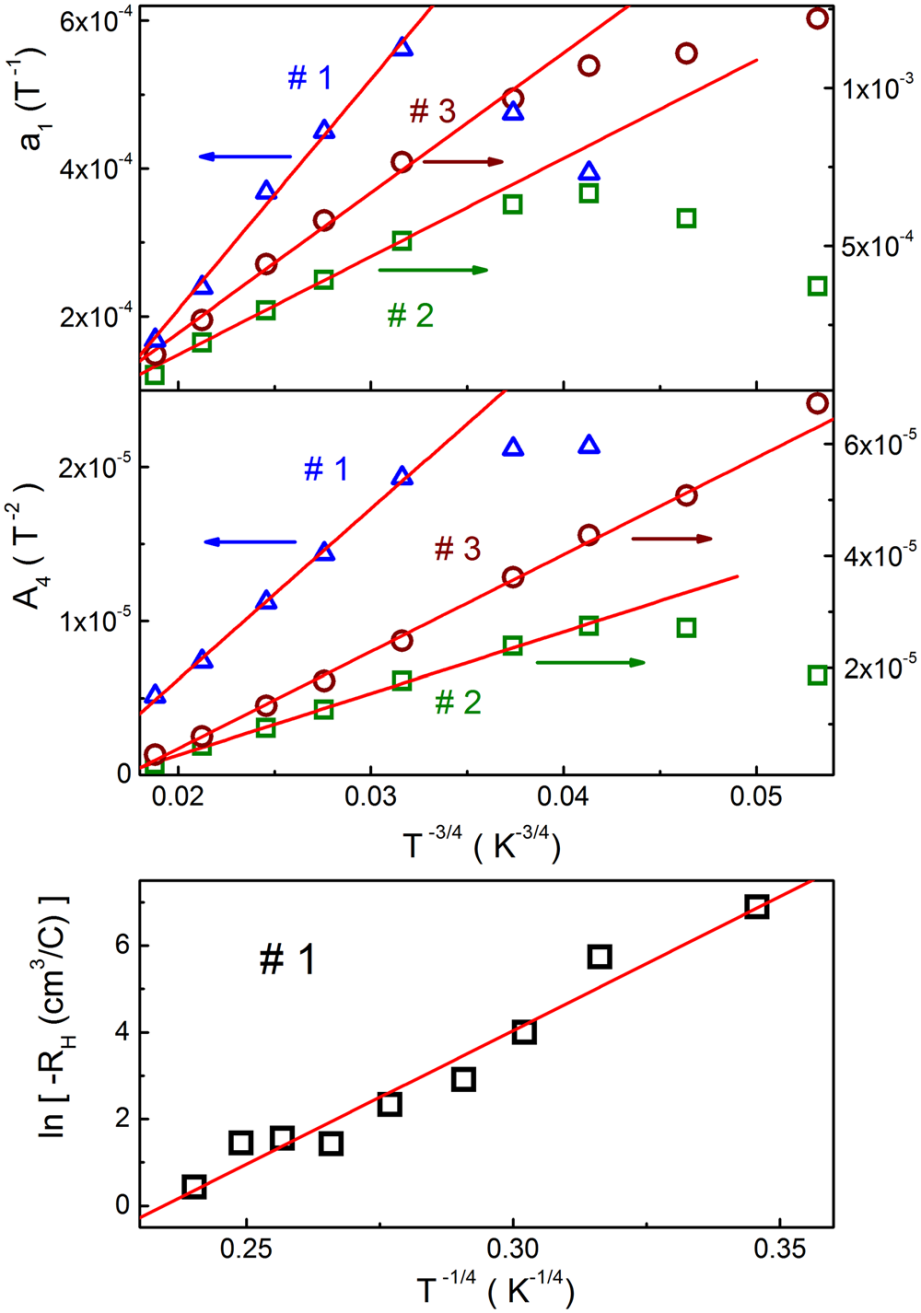
The plots of *a*_1_ vs. *T* ^−3/4^ (top panel), *A*_4_ vs. *T* ^−3/4^ (middle panel) and ln(−*R*_H_) vs. *T* ^−1/4^ (bottom panel). The lines are linear fits.

As can be seen in Fig. 5, the procedure above can be done for the majority of the MR plots in Fig. 2, including those lying within the VRH interval Δ*T*_v_ (some of the data are not shown only for convenience), whereas the onset of the linear dependence, lying between *B*_ons_ ~5–12 T, can be found only approximately. However, the interval of linearity looks sufficient for a reasonably accurate determination of both parameters, *a*_1_(*T*) and *A*_4_(*T*), which are plotted in the top and middle panels of Fig. 6, respectively, as functions of *T* ^−3/4^. One can see a good linearity of both functions within a whole temperature interval ~100–200 K of the Mott VRH conduction regime (cf. Table 1), deviating from such behavior only below 80 K. The values of *A*_04_ were obtained with linear fits of the plots in the middle panel of Fig. 6 according to the expression of *A*_4_(*T*), given below Eq. (5). They are collected in Table 1.

Finally, existence of other nMR mechanisms, probably less universal than that discussed above, should be mentioned for the sake of completeness. In particular, we have tested the nMR models based on the Zeeman effect^75^ and on a possible sensitivity of DOS to the magnetic field^76^, as well as the Khosla-Fischer model, taking into account both nMR and pMR contributions and addressing nMR to the scattering of charge carriers by the localized magnetic moments^77^. Concerning the first two models^75, 76^, their application could not reproduce correctly either the field or the temperature dependences of nMR in our samples. Formally, our MR data can be reproduced with the last out of the models above^77^. However, this model requires a degeneracy of the carrier gas, which obviously does not take place in our samples contradicting to the strongly activated behavior of ρ(*T*) in Fig. 1. In addition, no evidence for existence of the localized magnetic moments (or any types of paramagnetic centers) have been obtained in CZGeS in any previous investigations. In particular, those of the magnetization^78, 79^ and the EPR^79^ measurements have established only a weak diamagnetic response, which does not permit to consider any model of nMR, based on localized magnetic moments, to be substantiated sufficiently.

### Determination of microscopic parameters

The first pair of the hole parameters, namely those of *a* and *g*(μ), can be found directly from the pair of Eqs (2) and (6). This is done using the data of *T*_0_ and *A*_04_ (which have been obtained from the slope of the plots in the middle panel of Fig. 6) collected in Table 1, irrespective to the details of the DOS model shown in Fig. 4. The values of *a* and *g*(μ) are collected in Table 2.

**Table 2:** The absolute (*a*) and relative (*a*/*a*_B_) values of the localization radius, the values of the DOS under different conditions [*g*(μ) and *g*_av_], the absolute (*N*_A_) and relative (*N*_A_/*N*_C_) values of the acceptor concentration.

Sample	*a* Å	*a*/*a*_B_	*g*(μ) 10^16^ meV^−1^ cm^−3^	*g*_av_ 10^16^ meV^−1^ cm^−3^	*N*_A_ 10^18^ cm^−2^	*N*_A_/*N*_C_
#1	9.6 ± 0.5	1.3 ± 0.1	2.0 ± 0.2	2.7 ± 0.3	7.8 ± 0.6	0.21 ± 0.02
#2	12.5 ± 0.6	1.7 ± 0.1	3.7 ± 0.3	7.6 ± 0.7	14.6 ± 0.7	0.40 ± 0.04
#3	11.4 ± 0.6	1.5 ± 0.1	1.6 ± 0.2	5.2 ± 0.5	12.6 ± 0.7	0.34 ± 0.03

However, further analysis designed for determination of such details of the hole spectrum, as positions of μ and *E*_c_, as well as those of *N*_A_, requires utilization of the DOS model, although without a detailed knowledge of its shape. At this point, the analysis above is valid for both possible positions of μ, namely, near −*W* and *W*, since a symmetrical DOS has been proposed. On the other hand, the arguments towards the strong compensation, formulated in Section “Density of the acceptor states”, still dictates the position of μ as shown in Fig. 4. Further argument for such a choice will be obtained below.

The general expression of the localization radius can be written in a form

10 $$a={a}_{B}{(1-{N}_{A}/{N}_{C})}^{-v},$$

where ν ≈ 1 is the critical index of the correlation length and *N*_c_ is the critical acceptor concentration^51–54^. The latter is connected to the Bohr radius with the universal Mott criterion, ${N}_{C}^{1/3}{a}_{{\rm{B}}}\approx 0.25$ ^51, 52^. With the value of *a*_B_ = 7.5 Å, calculated above, we obtain *N*_C_ ≈ 3.7 × 10^19^ cm^−3^. Moreover, *N*_A_ can be found with the expression

11 $${N}_{A}={N}_{C}[1-{(\frac{{k}_{{\rm{B}}}{T}_{0}g({\rm{\mu }}){a}_{{\rm{B}}}^{3}}{{\rm{\beta }}})}^{1/(3\nu )}],$$

obtained with Eq. (2) and (10). The data of *N*_A_ are collected in Table 2.

On the other hand, according to ref. 51, another expression of *a*, conformed with the energy scale of Fig. 4, can be written as

12 $$a={a}_{{\rm{B}}}{(1-\frac{W+\mu }{W+{E}_{c}})}^{-\nu },$$

where

13 $${E}_{{\rm{c}}}=-W+{V}_{0}^{2}/[4(z-1)J].$$

In Eq. (13), *V*_0_ ≈ 2 *W* is a typical width of a carrier potential energy, expanded due to the disorder, *z* = 6 is the coordination number and *J* = *J*_0_ exp(−*R*/*a*_B_) is the overlap integral^51^. Here, *R* = (4π*N*_A_/3)^−1/3^ is the half of the mean distance between acceptors, whereas the prefactor *J*_0_ for the case of the broad impurity (acceptor) band can be expressed as^41, 51^

14 $${J}_{0}=\frac{{e}^{2}}{{{\rm{\kappa }}}_{0}{a}_{{\rm{B}}}}[\frac{3}{2}(1+\frac{R}{{a}_{B}})+\frac{1}{6}{(\frac{R}{{a}_{B}})}^{2}].$$

The values of μ and *E*_c_ are obtained with Eqs (12–14), using the value of κ_0_ = 6.8 cited above, the value of *a*_B_ = 7.5 Å and those of *N*_A_ in Table 2, by variation of μ to fit the data of *a* in Table 2 with Eq. (6) explicitly for each sample. The data of *W* in Table 1 and *N*_A_ in Table 2 permitted evaluation of the average DOS values, *g*_av_ ≡ *N*_A_/(2 *W*), which are collected also in Table 2. For convenience, the values of μ and *E*_c_ are displayed in a separate Table 3 below (see section “Discussion”). Finally, the data of *E*_a_ have been calculated with Eq. (4), using those of μ and *E*_c_ (Table 3), and are collected in Table 3, too.

**Table 3:** The values of the Fermi energy (μ) and the mobility edge (*E*_c_), the activation energies (calculated, *E*_a_ = |μ − *E*_c_|, and experimental, *E*_a_^(ex)^), and the interval of activated conduction (Δ*T*_a_).

Sample	−μ meV	−*E*_c_ meV	*E*_a_ meV	*E*_a_^(ex)^ meV	Δ*T*_a_ K
#1	127 ± 8	55 ± 3	72 ± 8	78 ± 2	215–255
#2	88 ± 5	74 ± 4	14 ± 2	15.1 ± 0.5	20–40
#3	107 ± 6	81 ± 4	26 ± 3	25.6 ± 0.8	60–75

### Hall coefficient

The Hall effect in the domain of the VRH conduction has different nature, than in the case of the band conductivity over the delocalized states (or drift conduction), connected to the Lorenz force. Therefore, the sign of the Hall resistivity, ρ_H_, and of the Hall coefficient, *R*_H_, should not be obligatory the same as that of the charge carriers. Namely, it can be opposite to the sign of the thermopower, which has been observed in the amorphous, as well as in the doped crystalline semiconductors^54, 80–85^, including our case of CZGeS (see Fig. 3).

The early theories of the Hall effect under VRH conduction predicted the behavior of *R*_H_ (*T*) similar to ρ(*T*), given by Eq. (1), but with another (and much smaller) value of the characteristic temperature *T*_0H_ ≈ 0.15*T*_0_ due to the influence of the exponential dependence of the Hall mobility, μ_H_(*T*) = *R*_H_(*T*)/ρ(*T*). However, this prediction has not obtained a comprehensive experimental support, meaning observations of *R*_H_(*T*) with *T*_0H_ both smaller than *T*_0_ and close to *T*_0_ (see ref. 54 and references therein). Moreover, it has been even suggested in investigations of CuInSe_2_, that the dependence of *R*_H_(*T*) is not exponential at all, whereas the resistivity-like behavior with the same value of *T*_0_ should be addressed only to the Hall mobility^81, 82^.

On the other hand, further theoretical work, based on an explicit percolation approach to the treatment of the Hall effect^80, 83–85^. has given the evidence for only a weak (power-law) temperature dependence of μ_H_ (*T*) and for the exponential dependence of *R*_H_(*T*), same as that of ρ(*T*). Namely, according to the results of ref. 80, the temperature dependence of the Hall coefficient can be presented in the form

15 $${R}_{{\rm{H}}}(T)=C(T)\exp [{(\frac{{T}_{0{\rm{H}}}}{T})}^{1/4}],$$

where *T*_0H_ = *T*_0_ and the dependence of *C*(*T*) is quite weak (logarithmic).

As can be seen in Fig. 3, some scattering of the data points does not permit to establish deviations of ρ_H_(*B*) in # 1 from linearity unambiguously, excluding probably only the case of low fields at 70 K. Therefore, the plots in Fig. 3 have been fitted with the linear function within the whole interval of *B* to obtain *R*_H_(*T*), and the plots of ln(−*R*_H_) vs. *T* ^−1/4^, neglecting the very weak dependence of *C*(*T*), are presented in the bottom panel of Fig. 6. The linear fit of the data yields the value of *T*_0H_ = (1.5 ± 0.4)×10^7^ K.

## Discussion

The good linearity of the plots in Fig. 5, accompanied with the dependences of *a*_1_(*T*) and *A*_4_(*T*) in the top and middle panels of Fig. 6, respectively, provide a sufficient evidence of the interpretation of both nMR and pMR contributions to the total MR of our CZGeS samples, proposed in Section “Analysis of magnetoresistance”. In addition, the analysis given in previous Section permits a more straightforward evaluation of the critical field *B*_c_ according to Eq. (8), using the explicit values of *a* in Table 2, those of *T*_0_ in Table 1 and J_0_ = 3.0, 2.4 and 2.5 eV for #1, #2 and #3, respectively, following from Eq. (14). So, one can obtain the values of B_c_ ≈ 3–9 T, which are comparable but smaller than those of B_ons_ ≈ 5–12 T marking the onset of the linear behavior in Fig. 5. Such a relation between B_c_ and B_ons_ is in a reasonable agreement with the linear contribution of nMR, as discussed in Section “Analysis of magnetoresistance”. On the other hand, the deviations from linearity of the plots of A_4_ vs. *T* ^−3/4^ and especially those of a_1_ vs. *T* ^−3/4^ (see the top and middle panels of Fig. 6) already close to the lower border of the VRH conduction (i. e. below ~80 K) look too strong. This requires a special discussion. At this point, only the orbital interference nMR mechanism may be insufficient to account for all details of the temperature dependence of nMR in our samples. Namely, the violation of the linearity of the plots in Fig. 6 with lowering *T* may be connected also to importance of the spin disorder and spin correlation effects of the hopping electrons with decreasing temperature^86–88^. Indeed, the temperature dependence of nMR due to the spin effects above is determined by the ratio of τ/τ_s_, where τ and τ_s_ are the characteristic hopping time and the spin relaxation time, respectively, having non-trivial and quite different dependences on *T* ^87, 88^. Hence, the issue above is attributable also to the contribution of spin-disorder and spin-correlation effects, which are more important when *T* is decreased^86–88^. A similar situation with the anomalous temperature behavior of the nMR contribution has been observed recently in the magnetotransport of Cu_2_Zn(Sn_x_Ge_1-x_)Se_4_ single crystals^11^.

For the next, as follows from the data in Table 2, the small values of the ratio of *N*_A_/*N*_C_ ~0.2–0.4 agree completely with those of another ratio, *a*/*a*_B_ ~ 1.3–1.7 exceeding unity only slightly. As can be seen with Eq. (10), the values of both ratios above mean, that all the investigated CZGeS material lies relatively far from the metal-insulator transition. In addition, one can see in Table 2, that the values of *g*(μ) are comparable with the average DOS values, *g*_av_, but are systematically smaller. The comparability of *g*(μ) and *g*_av_ supports the consistence of our analysis in general, whereas the smallness of *g*(μ) with respect to *g*_av_ is evident from Fig. 4, provided that the position of μ is shifted towards one of the DOS edges.

Eventually, the shift of μ towards the left DOS edge, or −*W*, suggests that the degree of the compensation in our samples is sufficiently strong. The arguments for such a situations have been already given in Section “Density of the acceptor states”. In addition, the values of *E*_a_ have been evaluated in Section “Determination of microscopic parameters” above (see Table 3). Such values of *E*_a_ suggest existence of the intervals Δ*T*_a_ of the activated ρ(*T*) behavior, given by Eq. (3), which is not connected to the Mott VRH behavior within the corresponding interval Δ*T*_v_, but is due to the activation of the holes into the interval (−*E*_c_, *E*_c_) of the delocalized states of the acceptor band (see Section “Density of the acceptor states”, inset in the top panel and the bottom panel of Fig. 1). Namely, the relatively high value of *E*_a_ for #1 should shift Δ*T*_a_ above Δ*T*_v_, whereas small values of *E*_a_ for #2 and #3 suggest that Δ*T*_a_ should lie below Δ*T*_v_. The corresponding intervals Δ*T*_a_ have been found by the linearization of the plots in the inset to the top panel of Fig. 1 and in the bottom panel in Fig. 1, and are given in Table 3. The values of *E*_a_^(ex)^, obtained from the slopes of the linear fits of these plots, are displayed in Table 3, too. One can see that, indeed, Δ*T*_a_ for #1 lies above Δ *T*_v_, while those for #2 and #3 lie below Δ*T*_v_. Such behavior is accompanied with the experimental values, *E*_a_^(ex)^, lying quite close to those of *E*_a_, evaluated in previous Section (see Table 3). This supports completely the position of μ in Fig. 3, as well as clarifies the nature of the charge transfer in CZGeS outside the Mott VRH interval, indicating a sufficiently strong degree of the compensation *K* in our material, as has been supposed above. On the other hand, the too strong compensation does not favor the nMR effect in general^39, 65^, which in turn may limit the value of *K* from above.

Finally, one can see that the Hall coefficient (bottom panel of Fig. 6) vary with *T* sufficiently close to the law of Eq. (15). In addition, the value of *T*_0H_ = (1.5 ± 0.4) × 10^7^ K is close to that of *T*_0_ = 1.43 × 10^7^ K for #1, following from the ρ(*T*) dependence of Eq. (1). This suggests a reasonable agreement with the percolation model of the Hall effect in the domain of the Mott VRH conduction^80^. However, some deviations from the model of ref. 80, including a possible exponential contribution to the Hall mobility, cannot be excluded due to a rather high error of *T*_0H_.

From the presented results, it is evident that the charge transport mechanisms in CZGeS have a complicated nature. In particular, this leads to observations of the VRH conductivity at unusually high temperatures, including the nMR and the pMR contributions in non-zero magnetic field, as well as to the non-conventional Hall effect. All these findings, including the variation of the macroscopic and microscopic parameters from sample to sample, should be considered anyway during the production of CZGeS based optoelectronic devices. Particularly for the solar cell use, the key problem of this material is its high tolerance to the intrinsic defect formation. Confirmed deep position of defect acceptor levels, as well as their broadening into the quite wide acceptor bands, may have an important detrimental influence on the photo carriers recombination and the final device efficiency. Therefore, the growing conditions of the CZGeS absorber layer for the thin film solar cells should be optimized to prevent or at least minimize the formation of the deep defects. In addition, the non-conventional Hall effect, which does not have any direct connections to the hole concentration, should be taken into account with a certain cautions for the characterization of CZGeS materials.

## Conclusions

We have investigated the resistivity, the magnetoresistance and the Hall effect in p-Cu_2_ZnGeS_4_ single crystals in pulsed magnetic fields up to 20 T. The dependence of ρ(*T*) in zero magnetic field is described by the Mott VRH charge transfer mechanism within a broad temperature interval of ~100–200 K. Magnetoresistance contains the positive and negative components, which are interpreted by the common reasons of doped semiconductors, including the shrinkage of the impurity wave functions by the magnetic field and the damping of the electron interference effects in the VRH hopping conduction regime, respectively. On the other hand, the joint analysis of the resistivity and MR data has yielded a series of important electronic parameters and permitted specification of the CZGeS conductivity mechanisms outside the temperature intervals of the Mott VRH conduction. The Hall coefficient is negative, exhibiting an exponential dependence on temperature, which is quite close to that of ρ(*T*), as typical of the Hall effect in the domain of the VRH charge transfer.

## Materials and Methods

Single crystals of CZGeS were grown by a chemical vapor transport using iodine as a transport agent. The growing process was performed in the vertical two-zone furnace with 850 °C in the evaporation zone and 800 °C in the growth zone. More details of the crystal growth process could be found elsewhere^20^.

Three CZGeS samples were selected for detailed investigations by a most convenient shape for electrical measurements. Chemical composition of the samples was determined by the X-ray fluorescence method, performed in three different points of each sample. The obtained data do not vary significantly from point to point, and the mean values are presented in Table 4. It can be seen, that all samples have the composition close to stoichiometry, exhibiting a slight Cu and Zn excess for #2 and some Cu and Zn deficient for #3 and #1. A part of the obtained material was grounded for the X-ray diffraction analysis, which showed the wurtzstannite structure of all the samples^20^.

**Table 4:** Chemical composition of the Cu_2_ZnGeS_4_ single crystals.

Sample	Cu at.%	Zn at.%	Ge at.%	Cu/(Zn + Ge)	Zn/Ge
#1	24.1	12.6	13.3	0.93	0.95
#2	25.4	13.0	11.6	1.03	1.12
#3	24.1	12.4	13.5	0.93	0.92

The hot point probe method, addressed to the thermopower measurements, showed *p*-type conductivity in all samples. Six indium contacts were made in each sample. The resistivity, ρ(*T*), was measured with a standard dc method, and MR was investigated in the pulsed magnetic field with *B* up to 20 T (see ref. 39 for the device details). The sample was fixed on the holder and inserted inside a cryostat. A filling helium cryostat was used to vary the sample temperature in the range of 20–300 K. A finger of the cryostat, containing the sample, was installed inside a pulsed solenoid. The main sample plate was oriented perpendicular to the direction of the magnetic field. A measurement at a certain *T* was performed by increasing and decreasing *B* from 0 up to the values between ~0.5–20 T, with subsequent changing of the field polarity. The results with the opposite polarities were averaged with a special software, to obtain the parallel (ρ) and the perpendicular (ρ_H_) components and to avoid their mutual influences. Eventually, series of above measurements was integrated to obtain the dependence of ρ(*B*) and ρ_H_(*B*) over the whole field diapason. Finally, it should be noted that the measurements at lower temperatures were hindered by the high sample resistance, which exceeded somewhat the installation limits.
